# Network Regulation of microRNA Biogenesis and Target Interaction

**DOI:** 10.3390/cells12020306

**Published:** 2023-01-13

**Authors:** Shintaro Komatsu, Hiroki Kitai, Hiroshi I. Suzuki

**Affiliations:** 1 Division of Molecular Oncology, Center for Neurological Diseases and Cancer, Nagoya University Graduate School of Medicine, Nagoya 466-8550, Japan; 2 Department of Nephrology, Nagoya University Graduate School of Medicine, Nagoya 466-8550, Japan; 3 Division of Nephrology, Department of Medicine, Duke University School of Medicine, Durham, NC 27710, USA; 4 Institute for Glyco-Core Research (iGCORE), Nagoya University, Nagoya 464-8601, Japan; 5 Center for One Medicine Innovative Translational Research, Gifu University Institute for Advanced Study, Gifu 501-1193, Japan

**Keywords:** microRNA, biogenesis, target-mediated miRNA degradation, cotargeting

## Abstract

MicroRNAs (miRNAs) are versatile, post-transcriptional regulators of gene expression. Canonical miRNAs are generated through the two-step DROSHA- and DICER-mediated processing of primary miRNA (pri-miRNA) transcripts with optimal or suboptimal features for DROSHA and DICER cleavage and loading into Argonaute (AGO) proteins, whereas multiple hairpin-structured RNAs are encoded in the genome and could be a source of non-canonical miRNAs. Recent advances in miRNA biogenesis research have revealed details of the structural basis of miRNA processing and cluster assistance mechanisms that facilitate the processing of suboptimal hairpins encoded together with optimal hairpins in polycistronic pri-miRNAs. In addition, a deeper investigation of miRNA–target interaction has provided insights into the complexity of target recognition with distinct outcomes, including target-mediated miRNA degradation (TDMD) and cooperation in target regulation by multiple miRNAs. Therefore, the coordinated or network regulation of both miRNA biogenesis and miRNA–target interaction is prevalent in miRNA biology. Alongside recent advances in the mechanistic investigation of miRNA functions, this review summarizes recent findings regarding the ordered regulation of miRNA biogenesis and miRNA–target interaction.

## 1. Introduction

MicroRNAs (miRNAs) are versatile post-transcriptional regulators of gene expression [1,2,3]. MiRNAs are small non-coding RNAs (ncRNAs), approximately 22 nt in length, which mainly utilize the 5′ seed sequences (nucleotides 2–7) to recognize diverse target mRNAs and direct them for suppression. Because of the dependency on short seed sequences for target recognition, one miRNA affects many genes, and one gene can be affected by multiple miRNAs. In particular, the 3′-UTRs of each mRNA contain multiple miRNA target sites and undergo complex and context-dependent regulation by multiple miRNAs. In addition, related miRNA families are frequently encoded together in polycistronic miRNAs [4]. The coordinated or network regulation of both miRNA biogenesis and miRNA–target interaction is prevalent in miRNA biology. Alongside the expansion of mechanistic insights into miRNA biology, this review summarizes recent findings regarding the ordered regulation of miRNA biogenesis and miRNA–target interaction.

## 2. Biogenesis of Canonical miRNAs

**Figure 1:**
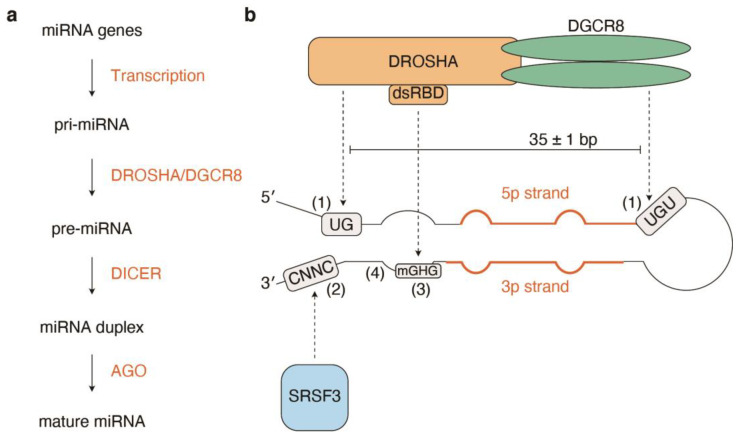
Structural basis of efficient pri-miRNA processing. (**a**) Outline of the canonical miRNA biogenesis. (**b**) The structure of pri-miRNA and related molecules are demonstrated. The numbers in parentheses indicate the features of the miRNA precursor sequences contributing to efficient pri-miRNA processing. (1) UG and UGU motifs are recognized by DROSHA and DGCR8, respectively. Thus, the DROSHA–DGCR8 complex contributes to the correct measurement of the miRNA basal stem length. (2) A CNNF motif is bound by SRSF3. SRSF3s have multifaceted roles in miRNA processing by facilitating pri-miRNA processing efficiency, modulating alternative processing, and preventing 5p-nick processing and inverse processing. (3) An mGHG motif is bound by the dsRBD of DROSHA and helps DROSHA identify the miRNA cleavage site. (4) The large-sized asymmetric internal loops, i.e., large-sized mismatches, inhibit the cleavage of the 3p strand and therefore downregulate miRNA expression. Pri-miRNA, primary miRNA; mGHG, mismatched GHG; dsRBD, double-stranded RNA binding domain.

The biogenesis of canonical and non-canonical miRNAs has been systematically and quantitatively characterized in many studies (Figure 1a) [1,2,3]. Canonical miRNAs in animals are transcribed by RNA polymerase II as long primary miRNA transcripts (pri-miRNAs). The highly active transcription of cell type-specific miRNAs via super-enhancers contributes to highly biased expression patterns of miRNAs, where a small subset of miRNAs dominates miRNA expression and function [5]. Hairpin structures within pri-miRNAs are cleaved by DROSHA and DGCR8 endonuclease complexes, yielding hairpin RNAs (precursor miRNAs, pre-miRNAs). The export of pre-miRNAs from the nucleus to the cytoplasm is mediated by exportin-5 (XPO5) and RAN-GTP, while the presence of other export mechanisms has been suggested [6]. In the cytoplasm, pre-miRNAs are further processed by DICER endonucleases to yield miRNA duplexes [7]. The miRNA duplex is loaded into Argonaute (AGO) proteins (AGO1–AGO4 in mammals). Once loaded, only one strand, termed the guide strand, whose 5’-nucleotide interacts with the MID domain of AGO proteins, is retained to form the final AGO–miRNA complex, termed the RNA-induced silencing complex (RISC). The choice of the guide strand depends on the identity of the 5′-nucleotide and the thermodynamic stability of the two ends of the miRNA duplex; a strand with 5′-uridine or 5′-adenosine and thermodynamically unstable 5′ ends is preferred [8,9,10,11]. The resultant RISC binds to target mRNAs mainly through sequence complementarity between seed sequences and target sites within mRNAs. TNRC6 (GW182) proteins, interacting partners of AGO, play important roles in target repression by interacting with the poly(A)-binding protein and recruiting the PAN2-PAN3 and the CCR4-NOT deadenylation complexes to the target mRNAs. Target recognition via AGO2 accompanies stepwise conformational changes in the AGO2 structure [12,13]. In the following sections, recent findings regarding the structural basis of pri-miRNA processing and “cluster assistance” mechanisms are summarized.

## 3. Structural Basis of Pri-miRNA Processing

Efficient pri-miRNA processing requires several pri-miRNA sequence features, including a narrow range of tolerable pri-miRNA stem lengths (35 ± 1 base pairs), a CNNC motif (SRp20/SRSF3-binding motif) 16–18 bp downstream of the DROSHA processing site, a UG motif at the base of the pri-miRNA hairpin, a mismatched GHG motif in the basal stem region, a stable lower basal stem structure (typically less than four mismatches), and a UGU(GUG) motif in the apical loop (Figure 1b) [14,15,16,17]. These features are highly conserved among animals [18]. Recent studies have provided mechanistic insights into these sequence features. DROSHA and DGCR8 form the heterotrimeric DROSHA–DGCR8 complex, consisting of one DROSHA and two DGCR8; the heterotrimeric complex recognizes a basal UG motif and an apical UGU motif via DROSHA and DGCR8, respectively [18,19]. This model is supported by recently solved cryo-EM structures of human DROSHA and DGCR8, which show that a molecular ruler consisting of two double-stranded RNA binding domains (dsRBDs) from DROSHA and DGCR8 is important for the measurement of pri-miRNA stem lengths between two dsRNA–single-stranded RNA (ssRNA) junctions [20,21].

For some miRNAs, the position of the mismatched GHG motif determines the DROSHA cleavage site [22]. The dsRBD domain of DROSHA recognizes a mismatched GHG motif to place the catalytic center in an appropriate position [22]. This supports the microprocessor cleavage precision [17,22]. In addition, a recent report described that the asymmetric internal loop in the lower stem of pri-miRNAs differentially affects the cleavage of pri-miRNAs on each strand [23]. The large asymmetric internal loop, that is, large mismatches, inhibits 3p strand cleavage, leading to a single cleavage on the 5p strand (5p-nick processing) and decreased miRNA expression [23]. Therefore, lower basal stem stability is associated with high miRNA expression levels [17,23]. In addition, the profiling of chromatin-associated pri-miRNAs supports the importance of more stable lower basal stems for pri-miRNA processing [17].

A recent high-throughput analysis of in vitro DROSHA processing of human pri-miRNAs confirmed these findings and revealed that among 1881 human pri-miRNAs in miRbase v21, only 758 pri-miRNAs are confidently processed by DROSHA, while the majority may be non-canonical or false entries [16]. The study unveiled many alternative processing and unproductive cleavage events such as “nick” or “inverse” processing [16]. Consistent with the impact of the asymmetric internal loop on 5p and 3p strand cleavage, 5p-nick processing events are more prevalent than 3p-nick processing events [16]. SRSF3, a binding protein for a CNNC motif, has multifaceted roles in miRNA processing by (1) facilitating pri-miRNA processing efficiency, (2) modulating alternative processing, and (3) preventing 5p-nick and inverse processing [16]. Given that miRNAs without typical CNNC motifs are also affected by SRSF3, the binding specificity of SRSF3 appears to be extended to ensure productive pri-miRNA processing [16]. A similar high-throughput study also highlighted the importance of low Shannon entropy and apical loop U bases for efficient pri-miRNA processing [24].

## 4. Cluster Assistance in Pri-miRNA Processing

**Figure 2:**
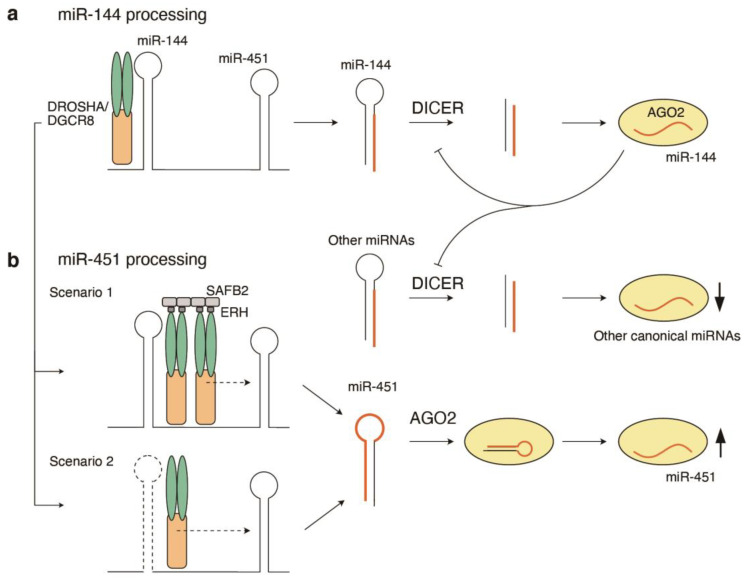
Cluster assistance mechanisms for processing of suboptimal pri-miRNAs. (**a**) The pri-miR-144 recruits DROSHA–DGCR8 and is processed by the canonical pathway. In erythropoiesis, miR-144 represses canonical miRNA biogenesis through DICER suppression, thereby making miR-451 the most abundant miRNA in erythrocytes. (**b**) For the processing of the suboptimal pri-miR-451, several scenarios have been proposed regarding the assistance from the optimal pri-miR-144. Scenario 1: DROSHA–DGCR8 recruitment to the optimal hairpins facilitates recruitment of another DROSHA–DGCR8 to the suboptimal ones through the dimerization properties of SAFB2 and ERH. Scenario 2: DROSHA–DGCR8 at the optimal hairpins is transferred to the suboptimal ones for continuous processing.

While the sequence features summarized above are important for the optimal processing of single pri-miRNAs, a fraction of endogenous pri-miRNAs are not necessarily optimal substrates of DROSHA. A series of recent studies revealed the interdependency of the processing of multiple pri-miRNA hairpins embedded in polycistronic pri-miRNA transcripts [25,26,27,28,29,30]. Pri-miRNAs often contain multiple miRNA hairpins, and this clustered arrangement can assist in the processing of otherwise defective or suboptimal hairpins. In particular, the presence of neighboring optimal pri-miRNA hairpins enhances the processing of suboptimal pri-miRNA hairpins to facilitate miRNA production [25,26,27,28,29]. This “cluster assistance” phenomenon is observed for diverse suboptimal pri-miRNA hairpins, including (1) miR-451 in the miR-144-451 cluster, which has a short loop and short stem, thereby relying on DICER-independent and AGO2-dependent maturation, and (2) miR-15a in the miR-15a-16-1 cluster, which has a large unpaired region in its lower stem [25,26,27,28,29]. Consistent with this, suboptimal canonical miRNA hairpins with a short loop preferentially reside in polycistronic pri-miRNAs [26]. Similarly, optimal miRNA hairpins enhance the processing of other miRNA hairpins within clustered viral miRNA transcripts in cis [30]. Importantly, the biological importance of the erythrocyte-specific miR-144-451 cluster is controlled by a sophisticated balance between the optimal miR-144 hairpin and suboptimal miR-451 hairpin (Figure 2) [25]. In erythropoiesis, miR-144 not only facilitates miR-451 production, but also represses canonical miRNA biogenesis through DICER suppression (Figure 2a), thereby making miR-451 the most abundant miRNA in erythrocytes [25].

Two accessory proteins of DROSHA–DGCR8 complexes, SAFB2 and ERH, have been reported as the mediators of “cluster assistance” [27,28,29]. A CRISPR/Cas9 screening of processing regulators of the suboptimal pri-miR-15a hairpin identified SAFB2 and ERH [29]. SAFB2 interacts with the DROSHA–DGCR8 complex and enables efficient processing of several clustered miRNAs [29]. ERH also interacts with the DROSHA–DGCR8 complex in 2:2 stoichiometry and facilitates the processing of suboptimal pri-miRNA hairpins [27,28,29]. Suppression of SAFB2 and ERH results in the downregulation of suboptimal miRNAs such as miR-15a, miR-92a, and miR-181b [27,28,29]. While the detailed mechanisms of “cluster assistance” remain elusive, several scenarios, which are not mutually exclusive, have been proposed (Figure 2b): (1) DROSHA–DGCR8 recruitment to the optimal hairpins facilitates another DROSHA–DGCR8 movement to the suboptimal ones through the dimerization (or multimerization) properties of SAFB2 and ERH; and (2) DROSHA–DGCR8 at the optimal hairpins is transferred to the suboptimal ones for continuous processing [26,27]. Consistent with both scenarios, the processing of clustered pri-miRNAs occurs sequentially [26,27,30]. As supporting evidence for the latter scenario, the insertion of additional suboptimal hairpins between miR-144 and miR-451 interferes with the miR-144-mediated enhancement of miR-451 processing [26]. The “cluster assistance” effects within polycistronic miRNAs may be much more complicated than for those within bicistronic miRNAs. The “cluster assistance” mechanism may also be important for the fidelity of the processing of suboptimal hairpins in vivo. Many suboptimal hairpins with unstable lower basal stems, such as miR-15a, miR-92a, miR-374b, and miR-181b, are subjected to 5p-nick processing [16,23] but are also supported with “cluster assistance” [26,27,29]. Given that the majority of miRNAs associated with nick processing in vitro do not undergo nick processing in vivo [16], the “cluster assistance” mechanism may contribute to the repression of non-canonical processing in vivo, together with multiple auxiliary factors, such as SRSF3. Many RNA-binding proteins (RBPs) are reported to regulate miRNA processing [31,32]. In addition, the stepwise or hierarchical pri-miRNA processing of oncogenic miR-17~92 is associated with the generation of processing intermediates, which are dynamically regulated during embryonic stem cell (ESC) differentiation or result in the downregulation of other polycistronic miRNAs [33,34].

## 5. Structural Basis of Pre-miRNA Processing

DICER, which belongs to the RNase III family, mediates pre-miRNA processing in the cytoplasm. DICER has two RNase III domains and one dsRBD located at the C-terminal region, the PAZ domain in the middle region, and three tandem RNA helicase domains (DExD/H domain) located at the N-terminal region, which are associated with pre-miRNA binding and cleavage. Among a series of functional and structural studies of DICER [7,35,36,37,38], a recent study demonstrated that the DExD/H domain has an ATP-independent essential structural role in mice and ensures the high fidelity of miRNA biogenesis in vivo [35]. The PAZ domain is thought to recognize the 5′- and 3′-ends of pre-miRNAs and determine their cleavage sites [39]. Consistent with this, a 3′-end mono-uridylation of a subset of pre-miRNAs with a shorter (1 nt) 3′ overhang promotes DICER processing [40]. In addition, apical loops or upper stem-loop regions (USL) of pre-miRNAs play important roles in DICER cleavage [41,42,43,44]. Recent high-throughput DICER cleavage assays revealed that a single-nucleotide bulge (22-bulge) facilitates the cleavage activity of DICER on shRNAs and human pre-miRNAs, and the stem lengths and defects in two RNase III domains and dsRBD differentially affect single cleavage events by DICER [45].

## 6. Inverse Regulation of miRNAs by Target RNAs: Target-Directed miRNA Degradation (TDMD)

**Figure 3:**
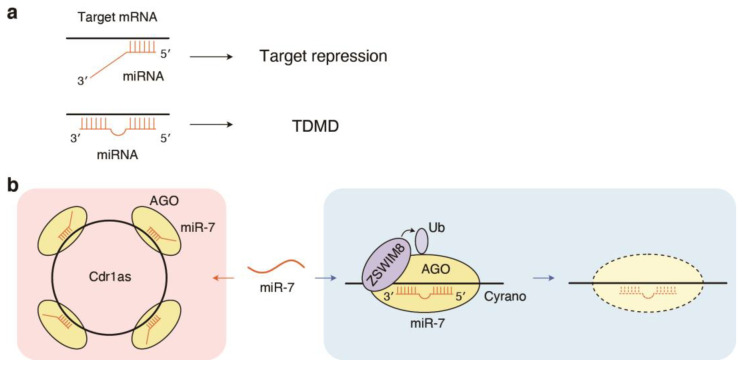
The miRNA decay by target-directed miRNA degradation (TDMD). (**a**) Distinct outcomes of target recognition: target repression vs. TDMD. (**b**) A long ncRNA Cyrano mediates TDMD of miR-7. ZSWIM8, an E3 ubiquitin ligase, binds to AGO2 and induces polyubiquitination and degradation of AGO2. By comparison, Cdr1as, a circular RNA with more than 70 binding sites for miR-7, interacts with miR-7 and prevents miR-7 from downregulation.

While the processing of pri-miRNAs and pre-miRNAs and the generation of miRNA duplexes occurs more rapidly than the generation of most mRNAs, miRNAs are generally very stable, with half-lives extending to days [46,47]. Nevertheless, the half-lives of individual miRNAs vary among cell types [47], and miRNAs are rapidly degraded in a context-dependent manner. In particular, highly complementary target RNAs promote miRNA decay through a process called target-directed miRNA degradation (TDMD) (Figure 3a) [48,49,50]. Initial examples of TDMD include the artificial target RNAs that induce 3′-end remodeling of small RNAs and the viral RNAs that induce miRNA decay [51,52]. A well-characterized example of endogenous TDMD includes the Cdr1as-miR-7-Cyrano and Nrep-miR-29b networks [53,54,55]. The Cdr1as-miR-7-Cyrano network consists of Cdr1as a circular RNA with more than 70 binding sites for miR-7, miR-7, and the long ncRNA Cyrano with an extensively paired site to miR-7 (Figure 3b) [53,54]. While Cyrano mediates the TDMD of miR-7, Cdr1as interacts with miR-7 and prevents miR-7 downregulation (Figure 3b) [53,54]. Cdr1as knockout mice display impaired sensorimotor gating, together with downregulation of miR-7 and upregulation of miR-7 targets in the brain [53]. The Nrep-miR-29b network consists of miR-29b and Nrep, with a near-perfect miR-29b target site in its 3′-UTR. Genetic disruption of the miR-29 site within Nrep in mice induces upregulation of cerebellar miR-29b and impairs coordination and motor learning [55]. Recent studies also highlighted the importance of TDMD and TDMD-like mechanisms in the proper development and developmental decay of miRNAs in *Drosophila* and *C. elegans* [56,57]. Potentially reflecting the importance of TDMD, 3′ non-seed base pairing were recently reported to contribute to the in vivo function of the evolutionarily conserved let-7a miRNA [58].

Mechanistic investigations of the well-established Cyrano-miR-7 TDMD pair recently identified the detailed molecular mechanisms of TDMD [59,60]. The target miRNAs of TDMD frequently exhibit 3′-to-5′ exonucleolytic shortening (trimming) and the addition of non-templated uridines or adenosines to the miRNA 3′ end (tailing). This is associated with conformational changes in AGO2, such as the dislodging of the 3’ end from the PAZ domain and the widening of the central cleft in AGO2 [61]. Furthermore, recent studies identified ZSWIM8 ubiquitin ligase as a direct mediator of TDMD [59,60]. Two groups employed CRISPR/Cas9 screening to identify a downstream mediator of Cyrano that destroys miR-7 and demonstrated that TDMD requires the ubiquitin E3 ligase ZSWIM8 (Figure 3b) [59,60]. When miR-7 interacts with Cyrano, ZSWIM8 binds to AGO2 and then destroys it, finally leading to the downregulation of miR-7 [59,60]. Conserved lysine(s) are critical for ZSWIM8-induced polyubiquitination and degradation of AGO2. In addition, ZSWIM8-mediated TDMD is independent of miRNA trimming and tailing. Recent computational approaches and systematic surveys of TDMD inducers using AGO-CLASH (cross-linking, ligation, and sequencing of miRNA-target RNA hybrids) have expanded the examples of TDMD triggers and suggested that TDMD triggers can assist the function of encoded proteins via the modulation of other miRNA targets [62,63]. It remains unclear how the properties of the target sites, especially 3′ complementarity, contribute to distinct outcomes, that is, target repression vs. TDMD.

## 7. miRNA Dosage Control by Fine-Tuning of miRNA Biogenesis Pathways

In contrast to TDMD, miRNA abundance is globally controlled by a fine regulation of miRNA biogenesis pathways [64]. As for DICER, miR-103/-107 and miR-144 were reported to induce global downregulation of canonical miRNAs through the repression of DICER [25,65]. As described earlier, regulation of DICER by miR-144 is important for miRNA regulation during erythropoiesis [25]. As for DROSHA–DGCR8, DGCR8 mRNAs have hairpin structures, which are destabilized by the DROSHA-DGCR8 complex, and thereby undergo post-transcriptional autoregulation [66]. DGCR8 is regulated by alternative transcription initiation [67]. Alternative transcription initiation using downstream promoters is enhanced during the differentiation of mouse embryonic stem cells and yields shorter DGCR8 mRNAs that do not have stem-loop structures [67]. Deletion of the stem-loop structures escapes autoregulation and results in an imbalanced DGCR8–DROSHA protein stoichiometry and irreversible aggregation of DROSHA–DGCR8 [67]. This reduces the efficiency of the pri-miRNA processing and the abundance of mature miRNAs, leading to de-repression of lipid metabolic mRNA targets [67]. This mechanism appears to be important in germ layer specification.

## 8. Impacts of Target Site Properties on Target Regulation

Target recognition by miRNAs mainly depends on the seed sequence of miRNAs [2,68]. The mechanistic understanding of target recognition by miRNAs has been improved by advances in computational approaches to predict miRNA targets, assessment of target regulation at RNA and/or translation levels upon perturbation of miRNAs, and high-throughput analyses of AGO2-bound RNAs, such as crosslinking-immunoprecipitation (CLIP) [1]. A recent systematic analysis using RNA Bind-n-Seq demonstrated relative binding affinities between AGO-miRNA complexes and seed sequences with various flanking sequences [69]. This approach confirmed the recognition of known canonical targets including 8mer, 7mer, and 6mer seed sites, and non-canonical target sites specific to each miRNA [69]. This study also showed that the affinities of canonical target sites vary among distinct miRNAs and that the dinucleotides flanking each site strongly influence the target site affinity [69]. In addition, the Halo-enhanced AGO2 pull-down (HEAP) method was recently developed to easily identify miRNA targets, particularly in vivo [70]. Computational and experimental approaches to decipher miRNA functions have been continuously developed [71,72,73].

Although target repression via non-canonical target sites is typically modest, non-canonical target sites appear to have context-dependent roles in miRNA function. A class of non-canonical target sites with extensive base-pairing at the 3′ site, but not the 5′ site, has been reported to function only in CDS regions [74]. Target repression through this class of target sites is independent of GW182 and involves translation repression with transient ribosome stalling but not mRNA destabilization [74]. While miRNAs frequently have non-templated 3′-uridine through TUT4/TUT7-mediated uridylation, a recent report described that complementary base pairing through 3′-uridine enables the repression of otherwise non-responsive seed-mismatch non-canonical targets [75].

Apart from TDMD triggers, binding affinities of target sites typically correlate with the strength of target repression: 8mer target sites exhibit more potent repression than 6 mer sites. Furthermore, a mixture of target sites with various binding affinities enables complex modes of biological regulation. For example, weaker miRNA targets are derepressed upon miRNA loss faster than stronger miRNA targets [76]. Conversely, weaker miRNA targets are repressed upon miRNA expression more slowly than stronger miRNA targets [76]. Therefore, target site affinity influences the temporal response to changes in cellular miRNA levels, explaining the cellular outcomes in mouse embryonic stem cells upon the global loss of miRNAs [76]. Furthermore, miRNAs modulate gene expression noise [77]. In this context, weak competing RNAs can introduce lower noise than strong competing RNAs [78]. Such target-dependent effects may influence the context-dependent biological roles of miRNAs, which can be reversed or switched by altering the target gene expression levels [79].

## 9. Expanded Mechanisms of Target Repression

The AGO-miRNA complex predominantly binds to the 3′ UTR of target mRNAs and induces target repression by deadenylating the poly(A) tails of mRNAs, repressing translation, and destabilizing the mRNAs [2,80]. A recent analysis demonstrated that miRNAs accelerate not only the deadenylation of their targets but also the decay of short-tailed target molecules [81]. This can explain the minimal effects of miRNAs on the steady-state poly(A)-tail lengths of their targets [81].

In many post-embryonic cells, mammalian miRNAs repress their targets predominantly by mRNA destabilization rather than translation repression [82,83,84]. A recent report described that translation is required for miRNA-dependent transcript destabilization [85]. Another report suggested that translating ribosomes play important roles in regulating the target site accessibility of target RNAs [86]. Live-cell single-molecule imaging of AGO2-dependent mRNA target silencing has revealed that ribosomes promote AGO2-target interactions and stimulate AGO2-dependent mRNA cleavage [86]. These effects are associated with the unmasking of target sites, which are frequently masked by interactions of the AGO2 target sequence with flanking mRNA sequences and combinations of multiple weak intramolecular mRNA interactions [86]. Supporting the importance of flanking sequences in miRNA target regulation, TDMD has also been reported to be influenced by the flanking sequences of TDMD sites [62]. Furthermore, a recent systematic analysis of the impacts of RBPs on miRNA targeting suggested that the binding of many RBPs close to an miRNA target site enhances miRNA targeting by unfolding mRNA secondary structures [87]. Widespread crosstalks between RBPs and miRNAs, including the “crosstalk with endogenous RBPs (ceRBP)” scenario, have also been described [88,89,90].

## 10. Significance of miRNA Cotargeting

**Figure 4:**
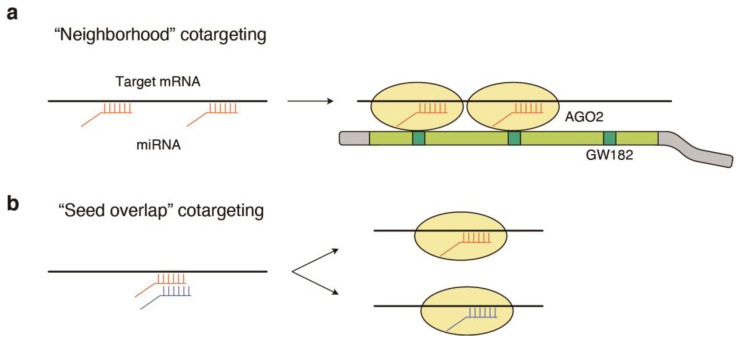
Distinct modes of miRNA cotargeting. (**a**) Closely spaced miRNA target sites can repress target mRNAs cooperatively. The mechanism of this “neighborhood” cotargeting can be explained by multivalent binding of GW182 proteins to multiple miRNA-AGO complexes. (**b**) In addition to “neighborhood” cotargeting, deeply conserved target sites frequently involve “seed overlap” miRNA cotargeting. While “seed overlap” target sites cannot be bound by two miRNAs simultaneously, “seed overlap” miRNA cotargeting increases susceptibility to downregulation by two miRNAs. The miRNA genes with extensive seed overlap are frequently organized in polycistronic clusters and/or distributed at multiple genomic loci.

Because many miRNA genes contain multiple miRNAs [4], multiple miRNAs are known to regulate target RNAs in a combinatorial manner. By doing so, potent inhibition can be achieved beyond the weak repression mediated by a single site. Earlier studies have shown that closely spaced miRNA target sites can repress targets cooperatively (“neighborhood” miRNA cotargeting) (Figure 4a) [91,92,93,94]. These synergistic effects can be explained by the multivalent binding of GW182 proteins to multiple miRNA-AGO complexes (Figure 4a) [95]. Cooperative regulation of biologically related target genes and pathways is frequently reported in the differentiation of various cell types, such as neuronal differentiation of embryonic stem cells, epithelial-mesenchymal transitions, and endocrine cell differentiation of pancreatic progenitor cells [96,97,98]. Furthermore, a recent genome-wide analysis of miRNA cotargeting demonstrated that hundreds of pairs of miRNAs share more mRNA targets than expected by chance, and that such miRNAs are particularly enriched in brain tissues, suggesting the importance of miRNA cotargeting in development, especially in the brain [99].

In addition, we recently reported the systematic characterization of another type of miRNA cotargeting, “seed overlap” miRNA cotargeting (Figure 4b) [100]. We previously reported that downregulation of two miRNAs, miR-128 and miR-148a, additively alters their target KLF4 via extensive overlapping of target sites, leading to enhancement of the inflammatory responses in plasmacytoid dendritic cells in the systemic lupus erythematosus (SLE) mouse model [100]. Although conserved “seed overlap” sites cannot be bound by two miRNAs simultaneously, in contrast to two closely spaced target sites, conserved “seed overlap” sites increase susceptibility to overall downregulation by two distinct miRNAs through additive recruitment of two miRNAs to these sites at the transcriptome level. Extensive “seed overlap” is a prevalent feature among broadly conserved miRNAs. Furthermore, miRNA genes with extensive seed overlap tended to have a higher probability of being miRNA cluster genes and being distributed at multiple loci, suggesting the importance of fine dosage control in this type of regulation. Systematic characterization of target sites further revealed unique evolutionary features of miRNA target sites. Highly conserved target sites of broadly conserved miRNAs are largely divided into two classes—those conserved among eutherian mammals and from humans to Coelacanth—and the latter, including KLF4-cotargeting sites, has a stronger association with both “seed overlap” and “neighborhood” miRNA cotargeting [100]. In addition, a deeply conserved miRNA target class has a higher probability of haplo-insufficient genes. Thus, the association of miRNA cotargeting with miRNA cluster genes, distribution at multiple genomic loci, and a high probability of haplo-insufficient genes suggests that fine-tuning the “dosage” of both miRNAs and target mRNAs is important for the network regulation of miRNA target genes [100].

## 11. Conclusions

The present review summarizes recent topics regarding the molecular basis of miRNA processing and target regulation, cluster assistance mechanisms of polycistronic pri-miRNA processing, TDMD, and miRNA cotargeting. Collectively, these findings suggest that coordinated or network regulation of both miRNA biogenesis and miRNA–target interaction is prevalent in miRNA biology. A better understanding of how miRNA genes evolve to adapt to miRNA processing and organize target networks will provide further insights into the development of miRNA-based diagnostic and therapeutic approaches.

## Data Availability

No new data were created or analyzed in this study. Data sharing is not applicable to this article.
